# SOCIAL ENGAGEMENT AND SUBJECTIVE COGNITIVE FUNCTION AMONG OLDER ADULTS: RURAL-URBAN DIFFERENCES

**DOI:** 10.1093/geroni/igac059.2949

**Published:** 2022-12-20

**Authors:** Alisha Thompson, Scott Wilks, Michelle Livermore

**Affiliations:** Louisiana State University, Baton Rouge, Louisiana, United States; Louisiana State University, Baton Rouge, Louisiana, United States; Louisiana State University, Baton Rouge, Louisiana, United States

## Abstract

Increasing age significantly relates with a decline in subjective cognitive function, while age and cognitive function decline strongly links with a diagnosis under the umbrella of Alzheimer’s disease and related dementias (ADRD). Recent literature points to social engagement as a potential mitigator to cognitive function decline. Social engagement often differs by geography of residence, with typically higher engagement among those in urban areas compared to rural counterparts. The purpose of this study was twofold: (a) to explore the relationship between social engagement and subjective cognitive function among community-dwelling older adults; and (b) to examine any disparity in social engagement apropos to rural vs. urban residency within this population. The theoretical framework, combining activity theory and cognitive reserve theory, guided the understanding of the relationship between social engagement and subjective cognitive function. A functionalist lens was included to explain rural-urban differences. Secondary data of community-dwelling older adults from AARP’s 2016 Social Engagement and Brain Health Survey (Nf746) were utilized. Results from descriptive analyses, correlations, and several regression models were reported. Bivariate regression models examined primary predictor variables – social engagement and geography of residence. Multivariate models examined sample characteristics, engagement, and geography of residence. Neither social engagement nor rural vs. urban residence achieved statistical significance in the models. Physical health, emotional wellbeing, marital status (identifying as married), and ethnicity (identifying as Black or African American) were positively, significantly associated with subjective cognitive function. Implications for community-dwelling older adults are discussed.

